# Supplementary material for: Are carbon emissions trading and green financial instruments synergistic? -Comprehensive quantitative research based on content analysis
Source: PLoS One. 2024 Mar 7;19(3):e0298601. doi: 10.1371/journal.pone.0298601 (PMC10919601; doi:10.1371/journal.pone.0298601)
Supplement: S1 Data — (ZIP) [file pone.0298601.s001.zip › Data/地级市Z_F工作报告.zip!/░▓╗╒/╬▀║■╩╨/└┤╫╘╣1⁄2╓┌║┼í╛┬φ┐╦╩2╛▌═°í┐_2015.docx]

——2015年1月20日在芜湖市第十五届人民代表大会第四次会议上

市长 潘朝晖

各位代表：

现在，我代表市人民政府，向大会报告工作，请予审议，并请市政协委员和其他列席人员提出意见。

一、2014年工作回顾

过去的一年，面对复杂多变的宏观经济环境，全市人民在中共芜湖市委的坚强领导下，全面深化改革，加快跨江发展，着力打造经济和城市两个升级版，实现了经济平稳较快发展、社会和谐进步。全市地区生产总值2307.9亿元（初步核算数，下同），比上年增长10.7%；居民人均可支配收入21947元，增长10.7%；财政收入426亿元，增长11.6%；城镇新增就业8万人；节能减排完成年度任务，单位生产总值能耗下降7.6%，PM10（可吸入颗粒物）年平均浓度下降2%。

一年来，我们坚持稳中求进工作总基调，统筹稳增长、促改革、调结构、惠民生、防风险，各项工作取得了新进展。

一是实施跨江发展战略。安徽省江北产业集中区总体规划（2013－2030年）基本完成，白茆镇划入市区。编制了大龙湾地区40平方公里中心区概念性城市设计，启动了新型城镇化示范区的规划和建设，博通创业园等项目开工。

坚持基础设施先行。合福铁路及无为站、南陵站施工进入扫尾阶段，长江公路二桥、北沿江高速公路、和谐大道等项目加快推进，商合杭铁路芜湖长江公铁大桥、庐铜铁路、通江大道改造及北延线、江北大道等工程开工。省江北产业集中区起步区服务配套功能进一步完善，累计入园产业项目56个，首批8家企业投产。市政务服务中心江北分中心投入运行。确定了江北新区供水、供气特许经营单位。开通了二坝、汤沟公交线路。无为经济开发区城东园区和高沟经济开发区道路、安置房、污水处理厂等基础设施加快建设。

实施对江北地区差别补助政策，市级拨付、提供投资担保25.5亿元，组建了注册资本16亿元的滨江建设发展公司。对皖B牌号7座以下小客车及公交车长江大桥通行费实行财政补贴。

二是扶持实体经济发展。认真落实中央和省稳增长、转方式、调结构的政策措施，出台了支持企业发展的19条意见。强化经济运行分析和监测，加大“一企一组一策”帮扶力度。落实税收政策优惠44.8亿元，减免行政事业性收费1.8亿元，对困难企业减缴缓缴社保费6.5亿元，发放稳岗和促进就业补贴2亿元。

加强重大项目调度。中兴通讯智能科技园、斐讯数据通讯产业园、东方雨虹建筑材料等项目开工，奇瑞汽车发动机和变速箱改扩建、海创新型节能板材、三山绿色铸锻工业园一期等项目投产。

加大金融支持实体经济力度。贷款余额增加232.6亿元，跨境融资和股权及关联方贷款净流入40.5亿元，完成直接融资202亿元。发挥国有担保机构、创业投资平台作用，对县区国有担保公司累计增资4亿元，缓解中小企业融资难，扶持科技型中小企业发展。楚江集团整体上市，长信科技等8家上市公司实施增发、并购，新增5家“新三板”、12家“四板”（区域性股权交易市场）挂牌企业。扬子农商行10亿元小微企业金融债和市宜居集团利用20亿元保险资金直投项目均为全省首单。

三是推动产业转型升级。以改造提升传统产业、聚焦培育战略性新兴产业、加快发展服务业为重点，提升产业竞争力。

壮大支柱产业和首位产业。汽车及零部件、材料、电子电器、电线电缆业共实现总产值3844.6亿元，增长10.4%。汽车及装备制造业实现产值2524.1亿元，增长13.1%。奇瑞汽车战略转型成效初显，新车型销量去年下半年同比增长2.2倍，占全部销量70%，单车均价提升40%。奇瑞路虎项目投产。鑫龙电器、鑫科新材料、弋江籽紫云英、新亚特电缆、华海电缆、恒升机床、江淮电缆、华星电缆、华亚塑胶成为中国驰名商标。

战略性新兴产业实现产值1533.4亿元，增长22.4%。推进机器人、新型显示两个国家战略性新兴产业集聚发展试点，首批16个项目完成投资44.8亿元。出台了机器人产业集聚发展若干政策，设立了哈工大、安徽工程大学2个机器人产业技术研究院，埃夫特万台主机、华研高压清洗机器人、翡叶伺服电机、固高驱动器等项目开工。构建平板显示产业链，长信科技显示屏减薄和触摸屏、华宇彩晶显示模组、德仓光电背光源模组等项目建成。建设通用航空产业园，中电科钻石飞机、奇瑞通航飞机发动机亮相珠海航展，新组建飞机设计研究院和飞机销售公司、航空营运公司。我市入选国家新能源汽车创新型产业集群试点，奇瑞eQ纯电动汽车上市，天弋能源锂电池等项目开工。推进循环经济产业园建设，汽车零部件集中回收体系初步建立。

服务业比重上升0.6个百分点。出台了现代服务业集聚区规划，三山物流园、芜湖县商贸物流园、大浦乡村文化旅游集聚区、市会展中心获批省级服务业集聚区。工信部电子五所芜湖实验室建成运行。鸿汇城、苏宁广场、华强广场、南翔万商物流城、麦德龙仓储超市开业，宇培速通物流、宝特铁路物流园、信安物流园项目开工，引进了中财物流信息港。中外运三山码头一期、朱家桥外贸码头二期投入使用。限额以上电子商务企业达16家，三只松鼠公司年销售额超10亿元，首批跨境贸易电子商务货物通关出口。跨境电商产业园首个项目投入运营。快递业收寄量增长1倍。我市成为国家电子商务、宽带中国示范城市和信息惠民试点城市。服务外包执行金额6.5亿美元，增长52%。10家企业入选全省民营文化企业100强。国家广告产业试点园区获批。方特三期、奇瑞日月星房车露营地、雨耕山文化创意产业园建成开业，方特五期开工。全市游客接待量2500万人次。成功举办第六届中国（芜湖）科普产品博览会、2014年中国体育文化·体育旅游博览会、第四届刘开渠奖国际雕塑大展等重大会展。

提升农业综合生产能力。粮食总产增长4.3%，规模以上农产品加工企业产值547亿元。“南陵圩猪”、“无为螃蟹”获农产品地理标志。繁昌县获批国家级农业综合标准化示范县。白茆镇被认定为省级现代农业示范区。以中联重机为龙头的农业装备产业跻身全国前列。

四是全面深化改革开放。推进行政体制改革。公布了市级政府权力清单、企业投资负面清单，市级政府权力事项减少到2565项，精简66%，其中行政审批仅保留166项，精简40%；市级核准投资项目减少33%。建立了260项事中事后监管制度。公布了市级涉企收费清单，收费项目精简到101项，精简49%。整合60个部门数据资源，138项行政管理事项实现在线办理。改革招标采购管理体制机制，实行管办分开，建设诚信评价体系和电子化交易平台。完成工商、质监行政管理体制调整和食品药品监督管理体制改革，县区整合设立市场监督管理局。深化公安改革，市区派出所由41个整合为24个，派出所警力占分局的93%。实施了市政、园林管养分离改革，组建市政、园林集团。实施注册资本登记制度改革，新设立公司7319户，增长73%。

深化金融改革，全国首批地方金融资产管理公司国厚公司、全省首家商业保理公司中安保理、全国首家住房金融公司惠居公司开业运营。扬子农商行投资5家村镇银行。加强政府性债务管理，开展存量债务清理处置，严控新增举债。建立了政府招商引资合同履约在线监管系统。深化土地制度改革，出台了经营性基础设施和社会事业国有土地有偿使用管理办法，在28个乡镇开展了农村土地承包经营权确权登记颁证试点，盘活工业用地7205亩，开发复垦整理新增耕地1.4万亩。

深化医药卫生改革，推行了城镇职工及新农合医保支付方式改革，全国首批基层卫生综合改革试点全面启动。深化教育改革，组建北师大基础教育实验小学、中学，优质学校办分校52个，建立学前教育、基础教育集团15个。加快社会服务管理信息化，易户网（一户一网站综合信息服务平台）、易企网（一企一网站综合信息服务与监管平台）上线运行。

扩大对内对外开放。出台以商招商实施办法，抓好重大项目和重点产业招商，亿元以上省外投资项目实际到位资金增长20%，实际吸收外商直接投资20亿美元。大陆电子三期、格力压缩机机体、中兴动力电池隔膜、首控高端机械装备、万向驱动轴、亚信软件外包基地、国购商贸城等重大项目签约。繁昌工业园区、鸠江经济开发区、三山经济开发区获批扩区。芜湖港总体规划基本完成，与上海港开展战略合作，在全省率先实施启运港退税政策，跨入亿吨大港行列，集装箱量突破40万标箱，增长40%。三山港区通过海关验收。长江经济带区域通关一体化、关检合作“三个一”（一次申报，一次查验，一次放行）模式顺利实施。实现进出口总额64.5亿美元，增长18.8%；其中出口49.7亿美元，增长26.6%。实施走出去战略取得突破，奇瑞巴西汽车、海螺印尼水泥项目投产。

五是加强城市建设。市委、市政府出台了关于提升城市品质的30条意见，促进城市建设与产业发展、人才集聚有效联动。坚持重大规划全覆盖，完成了城市排水防涝、城市供水备用水源、绿地系统、中心城区公共停车场等专项规划编制。出台了市政、园林设计和施工导则。

宁安城际铁路及火车站东站房、弋江站、繁昌西站主体工程基本完成。完善一级公路网，205国道南陵渡大桥改建、弋牧公路改建二期工程开工，318国道弋江至柯店段、329国道市区段改建工程完成。实施市区畅通工程，九华中路铁路立交、弋江路钱桥立交和黄山东路立交等项目开工，弋江桥和荆山桥改建、弋江路北延线等主体工程基本建成，华山路、十里牌立交建成通车。新购公交车248台，新增公交线路6条。建成了三山水厂和3座加气站。

将棚户区改造作为重大民生工程和发展工程强力推进，实施模拟征迁、自主征迁、市场化安置，让群众出棚即可进楼，完成棚改291万平方米。裕溪河治理工程开工，除险加固小型水库19座，治理中小河流5条，解决了15.1万农村居民饮水安全问题。新增绿地450万平方米、街头游园12个、绿道80公里。完成造林20万亩，建设森林长廊164公里。制订了节能减排低碳发展行动方案，实施节能减排项目110个，完成4台火电机组脱硫脱硝改造、11条水泥熟料生产线脱硝治理。淘汰落后水泥产能109万吨，整合关闭矿山20家，关闭全部烟花爆竹企业10家。落实秸秆禁烧、燃煤锅炉改造、淘汰黄标车等措施，防治大气污染。完成雨污分流改造项目25个，城东污水处理厂投入运营。新增光伏发电装机容量130兆瓦。完成3个城市主要出入口治理、44个“三线三边”整治、25个矿山地质环境治理项目。完成首批27个省级美好乡村示范村建设，南陵县、芜湖县分列沿江片区考核第1名、第2名。改造农村危房4572户。新建成7个国家级生态镇。经济技术开发区成为国家生态工业示范园区。改进城市管理考核机制，委托第三方开展城市文明程度指数测评。

六是鼓励创新创业。强化科技创新载体建设。长信科技、神剑新材料公司获批国家级企业技术中心。新增省级工程技术研究中心和企业技术中心26家、院士工作站6家、博士后工作站2家。组建重点产业技术研究院6家、省级企业孵化器5个、省级产业技术创新联盟3家。大学科技园获批为国家级科技企业孵化器。新增高新技术企业52家，培育科技“小巨人”企业80家。新增授权发明专利820件。我市成为全省首批创新型试点城市。

扶持青年创业、科技创业。出台了推进创业富民、建设创业特区的实施意见，大学生创业免抵押担保贴息贷款政策，新增发放小额担保贷款10亿元，安排创业富民扶持资金1.3亿元。建立了创业导师制度，开展了皖江青年科技创新创业大赛、大学生专利创新大赛、职工技术创新大赛等活动，实施高科技创业“雏鹰计划”，激发各类人才创新创业活力。

七是促进社会事业全面进步。实施了33项民生工程，完成投资83.5亿元。完成就业技能培训1.7万人，开发高校毕业生服务基层特定岗位330个，新增技能大师工作室2家。城镇登记失业率3.5%。城镇职工养老、医疗、失业、工伤、生育保险参保共达230万人次，增长5%。城乡居民养老保险、城镇居民医疗保险和新型农村合作医疗保险参保分别达162.8万人、104.2万人和218万人。2.2万人次享受大病医疗补充保险待遇3400余万元。实现省内异地就医双向联网实时结算。企业退休人员养老金月人均水平由1571元提高到1717元。为14.8万人发放低保金5.2亿元，低保标准提高10%以上。出台城乡医疗救助制度实施办法，为18.3万人次支出救助款9232万元。为生活无着人员发放社会救助金1744万元。新增住房公积金缴存人数5.4万人。1045户以船为家渔民上岸安居。为10881名农村原民办教师、离任村干部等群体发放了补助。建立了居家养老服务呼叫系统和信息平台，4个敬老院建设项目完工，21个社区日间照料中心投入使用。出台了公租房补贴细则，基本建成保障房5.5万套，4.1万户受益。

发展教育、卫生、文体等事业。新改建公办幼儿园11所，新认定民办普惠性幼儿园252所。完成55所学校标准化建设，建立名园长、名师、名班主任工作室37个。组建了芜湖高级职业技术学校、机械工程学校、科技工程学校、电缆工业学校、芜湖社区大学，安徽工程大学机电学院新芜校区、芜湖师范学校新校区投入使用。繁昌县、南陵县、芜湖县、弋江区、三山区通过义务教育发展基本均衡县（区）国家认定。全国（中西部）基础教育信息化应用现场会在芜召开。启动基层医疗卫生服务能力三年提升计划，培训全科医生100名。落实了“单独两孩”生育政策。市博物馆、规划展示馆和新四军七师纪念馆新馆开馆。繁昌县图书馆被文化部评为“全国最美基层图书馆”。启动历史文化名城保护规划编制，完成芜湖古城衙署前门整修。文学艺术、新闻出版、广播电视、地方志、科普、气象等工作得到加强，市档案馆晋升国家一级档案馆。我市在省第十三届运动会上取得金牌总数第二的好成绩。承办了四国青年男篮赛、全国青少年男子足球联赛、全国青年击剑锦标赛等赛事。

支持工会、共青团、妇联、红十字会、关工委等群团组织发挥作用。民族、宗教、外事、侨务、对台、妇女儿童、老龄、残疾人等工作进一步发展。高质量完成第三次经济普查登记。预防化解信访矛盾，群众利益诉求表达和回应渠道进一步畅通。完成了第九届村委会换届选举。镜湖区、鸠江区成为全国和谐社区建设示范区。完善了食品药品监督分级管理体制。安全生产形势进一步好转。严厉打击各类违法犯罪活动，深入推进“平安芜湖”建设，社会治安保持稳定。全民国防教育、国防动员、人民防空、双拥优抚工作进一步加强，对驻芜部队和民兵预备役建设的支持力度进一步加大。

去年，我们深入开展党的群众路线教育实践活动，践行“三严三实”，认真解决形式主义、官僚主义、享乐主义和奢靡之风方面存在的突出问题。坚持“机关联系基层、干部联系群众”，着力破解服务群众“最后一公里”问题。推行了以“微笑、真情、文明、敬业、满意”为主题的“亲切服务”。开通了“12345政府服务直通车”，统一办理和反馈市民诉求。举办新闻发布会16场，公开政务信息8万余条，办理回复网民咨询和市长信箱留言1.2万条。办理人大议案3件、建议196件和政协提案263件。制定了市政府工作规范，完善重大行政决策机制。强化审计监督和行政监察，开展了政风行风评议、民主评议科长、媒体问政活动，清理了领导干部在企业兼任职行为，加强廉政风险防控，对腐败行为依法严惩。

各位代表！过去一年的成绩来之不易。这是中共芜湖市委科学决策、坚强领导的结果，是市人大和市政协加强监督、鼎力支持的结果，是全市人民万众一心、团结奋斗的结果。我代表市人民政府，向各级人大代表、政协委员，向广大工人、农民、知识分子、干部、离退休老同志，向驻芜解放军指战员、武警官兵和政法干警，向各民主党派、各人民团体和各界人士，向关心支持芜湖发展的上级部门、兄弟市和海内外友好人士，向在芜湖创业发展的投资者、建设者，表示衷心的感谢！

我们也清醒认识到，经济社会发展中还有不少困难和问题。经济增速持续回调，新兴产业规模不大，服务业比重低，城市竞争力不够强；城市基础设施不够完善，功能和品质亟待提升；人才队伍规模不大，高层次人才紧缺；生态环境治理任务艰巨；各类风险防控压力加大；行政管理和政务服务水平有待提升，少数工作人员庸、懒、散现象突出。对此，我们一定高度重视，扎实有效解决问题，决不辜负人民的厚望。

二、2015年的主要工作

今年是全面深化改革的关键之年，是全面推进依法治市的开局之年，也是全面完成“十二五”规划的收官之年。当前，我国经济发展已进入新常态。必须清醒地看到，新常态下，传统产业产能普遍过剩，依靠一般性地承接产业转移和产能复制实现规模快速扩张的路子走不通了；房地产市场趋于平稳，区域竞争由资本争夺向人才和技术争夺蔓延，依靠简单化地铺摊子、上项目以及财政补贴实现规模快速扩张的路子也走不通了；环境资源约束不断增强，依靠低成本资源投入、不顾环境代价实现规模快速扩张的路子更走不通了。但是与此同时，传统行业加速并购集中，新兴产业快速崛起，新技术、新业态、新模式不断涌现，互联网对经济发展的影响日益深入，我们必须迅速调整发展思路、发展方式，准确把握新常态下经济发展新特征，打破简单铺摊子、上项目的传统思路，着力于传统产业转型升级和战略性新兴产业发展，着力于高科技、新业态、新模式等新的经济增长点培育，着力于以优秀人才为重点的各类创新创业资源的集聚，奋力走出一条产业层次高端化、城市品质高端化、人才结构高端化相互促进的内涵式城市发展路子。芜湖拥有良好的发展基础，又面临长江经济带建设的宝贵机遇，只要我们及时调整好发展思路和发展重点，抓住用好各种机遇，超前谋划，精准施策，持续打造经济和城市两个升级版，就完全有条件在新常态下展现新优势，实现更好更快发展和“弯道超越”。

今年政府工作的总体要求是：全面贯彻党的十八大、十八届三中、四中全会和中央、省市经济工作会议精神，坚持经济规模扩张与转型升级并举、城市跨江发展与品质提升并举、改革攻坚与扩大开放并举、推动创新与激励创业并举，全面推进依法行政，确保经济健康发展、社会和谐稳定、人民生活改善。

今年经济社会发展的主要预期目标是：地区生产总值比上年增长9.5%左右，居民收入、财政收入同步增长，城镇新增就业8万人，城镇登记失业率控制在4.5%以内，节能减排完成省政府下达任务。

围绕上述目标，重点做好以下工作：

（一）深化改革开放，为经济社会发展注入新动力

实施市、县区政府机构改革。创新行政审批体制机制，推行综合受理、网上审批。落实市、县区政府权力清单和责任清单、企业投资负面清单、涉企收费清单制度，建设政府权力项目库和权力运行、政务公开、法制监督、电子监察“四平台”，形成动态调整机制，探索建立基于信息化的事中事后监管体系。深化投资体制改革，推行PPP（政府和社会资本合作）投融资模式，引导社会资本进入轨道交通、市政设施、公共服务等领域。深化公共资源交易管理体制改革，完善诚信制度，建立市县一体化交易体系。实施公务用车制度改革。出台城市社区管理体制改革实施意见。改革供销社管理体制。深化商事制度改革。推进社会服务管理信息化，推动市县两级企业服务中心线上、线下常态化服务，实现涉企信息实时在线交换、涉企事项受办分离。推动政务信息化，逐步实现全市公务员“一张网”办公和在线考评。

深化预算管理制度改革，将政府的收入和支出全部纳入预算管理，推进预决算公开，提高财政支出绩效。严格规范政府性债务管理，探索建立政府投资项目民主决策、绩效评价、责任追究制度，切实防范债务风险。继续深化营业税改征增值税改革。出台农村金融服务综合改革方案、支持互联网金融产业发展的意见，争取设立民营银行、企业财务公司，推进小贷公司资产证券化试点，启动“金融市民卡”服务应用体系建设，争取3家企业首发上市，谋划发行项目收益债、中小企业私募债，争取直接融资200亿元以上。

深化医药卫生体制改革。出台引进社会资本参与公立医院改革的具体意见。完成国家基层卫生综合改革试点任务。实施基层医疗卫生服务能力三年提升计划，深化新农合支付方式改革，完善运行机制。实行绩效管理，调动医务人员积极性。

深化农村综合改革和土地管理改革。完成农村土地承包经营权确权登记颁证任务。实施全市不动产统一登记。推进农村产权交易体系建设试点。深化工业用地市场化配置改革，争取矿产资源节约集约开发利用试点。

开创对外开放新局面。以资本为纽带，加强芜湖港与相关港口的合作，实行优势互补、协同发展。促进省级以上开发区转型升级，推动出口加工区整合升级为综合保税区，提升通关一体化和投资贸易便利化水平。积极参与长三角城市群分工与合作，加快芜马同城化项目建设，推进芜亳合作共建。围绕主导产业，强化精准招商、产业链招商、以商招商，注重引进行业龙头和区域性、功能性总部机构。优化对外贸易结构，扩大汽车、机电产品出口和高端装备、先进技术、资源性产品进口，培育一批服务贸易企业。加大企业境外上市融资支持力度。加快实施走出去战略，引导企业开拓境外市场。

（二）聚力重大项目培育，增强产业核心竞争力

促进经济发展，产业是根本，项目是关键。

持之以恒抓好大项目、好项目的招商落地。按照产业转型升级的总体要求，继续集聚一批市场潜力大、带动能力强、科技含量高的重大项目。努力以最高效率、最优服务做好项目前期、融资、用地等关键环节的协调推进工作。力争新开工投资亿元以上项目400个，其中10亿元以上项目40个。推动捷瑞新能源汽车、南车浦镇庞巴迪运输系统、华电二期、华讯智慧教育产业园等项目开工，信义电子玻璃等项目建成。落实扶持中小微企业的政策措施，坚持扶优扶强，鼓励兼并重组，有序淘汰落后产能，提升民营经济素质。

推动传统产业向中高端迈进。壮大汽车及装备制造业，推动四大支柱产业与新兴产业融合发展，支持奇瑞、海螺、美的、新兴铸管等龙头企业做大做强。加强质量和品牌建设，创建全国质量强市示范城市。

聚焦突破战略性新兴产业。培育机器人、电子信息（新型显示）、新能源、通用航空等新经济增长点。突出发展工业机器人，并向工业自动化装备、智慧工厂和生活服务机器人等领域扩展。提升新型平板显示产业竞争力，大力发展电子信息产业，并向智能家居延伸。加快建设通用航空项目。抓紧新能源汽车的研发、生产和推广应用，配套建设充电设施。推进循环经济产业园项目建设，启动新材料产业培育发展规划。

大力发展服务业。建好省级现代服务业集聚区。着力突破现代物流、信息服务、商务服务、研发设计、检验检测等生产性服务业。发展临港产业，加快建设朱家桥综合港口物流园、三山公铁水联运大型物流枢纽、皖江航运服务业集聚区。推进电子商务示范城市建设，支持宇培吉品、三只松鼠、凡臣优品等电商企业发展，建立“阿里巴巴·芜湖产业带”平台，建好跨境电商产业园。建设皖南快递产业园。支持龙头企业在社区开设连锁便利店和直销菜店，培育3条特色商业街。实施养老健康家政、信息、旅游、住房、绿色、教育文化体育等6大领域消费工程。按照皖南国际文化旅游示范区建设规划，优化旅游产品结构和服务体系。推动文化旅游与相关产业融合发展，推进文化科技产业示范区、方特5A景区创建，建成非物质文化博览园、新华联海洋公园和老街一期、大砻坊工业设计文化产业园，加快建设“复兴之路”爱国主义教育基地，打造“欢乐芜湖”品牌，建设国家级旅游目的地。办好第四届中国动漫创意产业交易会、第三届中国电线电缆博览会。

积极发展现代农业。创新农业经营体系，鼓励发展种养大户、家庭农场、农民合作社，推进农超对接。发展高效生态农业、特色精品农业、都市型农业，提升农业产业化规模和档次，重点发展以食品加工和营销为龙头的产业化联合体。推进现代农业示范区建设。发展农业装备产业，支持中联重机做大做强，打造国际知名的农业装备产业集群。

（三）坚持跨江发展，着力提升城市品质

强化规划引领。开展港产城融合发展、城市景观风貌、城市规划区空间利用（城市增长边界）研究，启动城市单元整体性控制、内河岸线资源利用等规划，完成至2030年新一轮县城总体规划修编及公共服务设施等主要专业规划。推进县城与市区发展“等高对接”，城市宜居宜业品质与先进城市“等高对接”，争取有条件的县改市、改区。

强力推动跨江发展。围绕现代化大城市主城区的定位，完善江北新区经济社会发展、城乡建设、产业布局、土地利用、生态建设等规划，统筹生产生活生态功能，促进江南江北联动发展。推动江北产业集中区向产城一体、宜业宜居的现代化城区转型。推进大龙湾片区的基础设施建设和产业集聚。支持无为县加快城市化进程，支持无为经济开发区城东园区和高沟经济开发区转型发展，在重大项目布局上予以倾斜。加快商合杭铁路及长江公铁大桥、长江公路二桥、庐铜铁路、北沿江高速、和谐大道、江北大道、通江大道改造、芜合高速公路二坝（长安）互通立交、江北农贸市场等基础设施建设。推进宝能组团、海螺组团、修正健康饮品产业园、惠普全球汽车云产业基地、巨轮高端装备产业园、博通创业园、长联石油、无为信义光伏电站等项目建设。

打造综合交通枢纽，完善基础设施。建成宁安城际铁路及火车站东广场、合福客运专线，尽快迈入高铁时代。加快建设皖赣铁路扩能改造、芜申运河、铜南宣高速公路、205国道芜南段改造等工程。开工建设芜黄高速、沪渝高速公路峨山路互通工程，启动芜湖宣城民航机场建设。加强岸线资源管理和港口建设，发展多式联运，强化皖江航运枢纽地位。推进城市轨道交通项目前期工作，力争年底开工。启动城市交通提升六年行动计划，加快弋江路、峨山路快速化改造，建成7处行人过街通道。实施交通组织优化工程，建设智能交通系统。完善城东新区、县城及省级开发区生活服务设施，建设邻里中心示范点。开工建设市区备用水源，实施高层住宅二次供水改造。坚持统筹规划、成片改造棚户区，发挥改善群众住房条件、拉动投资和消费需求、带动相关产业发展、优化城市形态的综合效应，完成改造256万平方米。坚持服务与监管并重，优化产品结构，合理引导消费，促进房地产市场持续健康发展。加快建设青弋江分洪道、无为万亩圩口达标、澛港闸（桥），开工建设荆方大型排涝泵站、青弋江治理工程，整治城乡水系，确保安全度汛。

推行城市精细化管理。加强对城市生态网络空间的塑造和历史文化内涵的挖掘，重视人气商气培育，做好城市重要节点设计，有序引导地块出让，彰显“中西合璧、精致繁华”的城市特色。构建完善的城市路名牌、指示牌、门牌标识系统。提升城市绿化、美化、亮化水平。推动市县数字化城管系统数据资源共享。完善住宅区物业管理机制，提升物业服务水平。坚持集中整治和长效管理相结合，改善市容环境。

（四）激励大众创业、万众创新，建设人才特区、创业特区

继续实施人才引进培育、人才创新创业、人才平台建设和人才服务提升工程，面向全球广泛集聚各类人才，广泛吸引企业高管、技术人才、大学生、高校教师来芜创业，争取新引进高层次人才团队30个。打造国家级大学生创业孵化基地、留学归国人员创业园、省级青年创业园等创业平台，推动校地创业孵化合作，积极举办各类创业大赛、创业项目推介会和创业项目成果展。完善创业政策体系，落实免费提供创业场地、开办费补贴、水电费补贴、税费减免、小额担保贷款、政府采购、公租房房租补贴、就业奖励等措施，大力扶持高科技创业活动和新业态培育，促进高校毕业生就业创业。加快建立产业投资基金、政策性担保、财政补贴一体化的创业支持体系，继续在落户、就医、子女上学、购房等方面给予最大便利，营造全社会尊重人才、支持创业的良好氛围。

争创国家创新型试点城市。积极落实各级鼓励创新的政策，完善我市配套措施。统筹推进省级以上企业技术中心、工程技术研究中心、实验室、产业技术研究院、院士工作站等创新平台建设，打造共性技术研发平台，争取新增院士工作站3家、省级以上研发机构20家、产业技术研究院4家、博士后科研工作站2家。推动产学研深度结合，鼓励企业建设研发机构，鼓励高校院所设立技术转移机构和成果产业化基地，加快导入优质科技资源，加强协同创新。争取新增科技型中小企业300家。鼓励专利创造和运用，推动专利发展向质量效益型转变。

（五）保护生态环境，建设美好家园

实施一批减排重点项目。治理钢铁、水泥、电力、化工等重点行业污染，对重污染企业实行搬迁改造。推进省级以上开发区循环化改造和企业减排技术改造。坚决落实大气污染综合整治行动计划。抓好饮用水源地综合整治。加快建设餐厨废弃物和污水处理设施，实行市区污水管网统一管理。

实施90个节能重点项目。加强能源消费增量和消费强度控制，实施节能技术改造。推进中电国际（芜湖）环保电厂、无为丰原生物质电厂、华电福新能源风电场、绿洲垃圾焚烧发电厂改扩建等项目建设，加快创建国家新能源示范城市。强化土地利用动态巡查和用地监管，开展低效和闲置土地清理，盘活存量土地资源，提高土地节约集约利用水平。实行最严格的水资源管理制度。积极推行绿色建筑。

实施园林提升工程，新增绿地400万平方米，新建绿道80公里，建设管理好神山公园。推进森林增长工程，实行新增林地往市区、镇区和居民点集中，完成造林11万亩。支持芜湖县创建国家园林县城，支持繁昌县实施生态强县工程，支持芜湖县、南陵县争创国家级生态县。科学有序推进美好乡村建设和“三线三边”环境治理。推进全国水生态文明城市建设试点和国家卫生城市、国家环境保护模范城市、国家生态园林城市创建工作。

（六）保障和改善民生，促进社会和谐

计划投入91.6亿元，实施33项民生工程，进一步提升民生工程管理水平。

继续打造教育名城和皖江医疗中心。鼓励优质学校通过办分校、一校多区等方式，扩大优质教育资源共享面，推进基础教育均衡发展。力争新增教育集团5个，新增优质学校办分校（分园）5个，新增结对帮扶学校（幼儿园）50对。支持在芜高校发展。推进教育信息化，实现90%义务教育学校“班班通”。大力引进高水平教育医疗资源。优化医疗卫生设施布局，改善基本公共卫生服务。落实国家生育政策，改进计生服务。

完善就业和社会保障服务体系。开展实名制就业帮扶活动，着力解决高校毕业生、退役士兵、就业困难人员、零就业家庭就业问题。建设人力资源公共实训基地，推进校企合作和高校就业联盟工作。扩大社会保障覆盖面，完善城乡居民基本养老保险制度。规范城乡低保管理。重视做好扶贫工作。鼓励非公有制企业建立住房公积金制度。

推动文化繁荣发展。深入开展中国梦、中国道路、社会主义核心价值观的宣传教育，凝聚全市人民力量。深化文化体制改革。完善城乡文化设施，提升公共文化服务质量。加强文化遗产的传承、保护和利用。打造文艺精品。广泛开展群众文化活动。推进数字档案馆建设，发展地方志事业。推进传统媒体与新兴媒体融合发展。深入开展文明创建活动。加强征信系统建设，健全诚信“红黑榜”制度，培育诚信文化。

统筹发展各项社会事业。促进群众体育和竞技体育全面发展，完善体育场馆运营机制。加强民族、宗教、外事、侨务、对台工作，完成全国城市少数民族流动人口服务管理体系建设。发展妇女儿童事业。构建以居家养老为主的养老服务体系，鼓励社会投资养老服务业，探索公办养老服务机构“公建民营”管理模式。提升残疾人保障水平。开展环境气象监测与服务，启动防洪保安气象工程。

推进法治社会建设。完成“六五”普法任务，引导群众依法表达诉求、依法维护权益、依法履行权利和义务，在全社会树立起法律的权威。推进政社分开，引导社会组织在人员、场地、财力等方面与行政机关脱钩，更好发挥社会组织的作用。推进城乡公共法律服务体系建设，加强困难群众法律援助。开展“信访法治建设年”活动，坚持诉访分离，实行逐级走访，推行阳光信访，提高初信初访一次性办结率。推进安全监管责任体系建设，深化安全隐患排查治理。强化食品药品安全监管，完善从生产到消费的全程监管机制。完善应急预案管理，提高防灾减灾、应急保障能力。加强对社区矫正对象及特殊人群管理。开展“守护平安”系列行动，加快治安防控体系建设，提升社会治安水平，确保社会安定有序。深入推进综治维稳信访工作中心（站）和为民服务中心（工作站）规范化建设。增强全民国防、人防意识，支持驻芜部队建设，推进军民融合深度发展。开展双拥共建活动，落实优抚安置政策，争创新一轮全国“双拥模范城”。

三、加快建设法治政府

适应新常态，建设新芜湖，对政府工作提出了更高要求。我们必须自觉接受党的领导，忠实履行宪法和法律赋予的职责，把政府的一切权力和行为纳入法治轨道，加快建设职能科学、权责法定、执法严明、公开公正、廉洁高效、守法诚信的法治政府。

依法履行政府职能。健全科学民主依法决策机制，把公众参与、专家论证、风险评估、合法性审查、集体讨论决定作为重大行政决策的法定程序。健全重大决策终身责任追究制度及责任倒查机制。自觉接受人大及其常委会的法律监督、工作监督和政协的民主监督，重大决策出台前按程序向市委汇报、向人大报告、与政协协商，认真办好人大代表议案、建议和政协建议案、提案。深入调研、系统谋划、科学论证，高质量完成“十三五”规划纲要编制。创新政府履行职能方式方法，实现政府管理方式从注重事前审批向注重事中事后监管转变。完善政府购买公共服务机制，推动公益服务项目专业化运作。

深化执法体制改革。整合执法部门监管力量，充实基层人员，实行重心下移。探索综合监管、分类执法的模式。完善网格化管理机制，对不同的监管对象建立不同的监管手册，对网格内各类监管对象实行综合检查，检查结果上传统一信息平台，由有权的执法部门分别调查处理。建立执法全过程记录制度，推行行政执法公示制度，规范执法自由裁量权。全面落实行政执法责任制，加强对行政执法的监督。

提升政务服务效能。推动政务服务、人力资源服务、住房公积金管理、公共资源交易、房地产交易、社会化服务协调、企业服务、出入境管理等8家服务机构集中入驻“市民服务中心”。打造居民生活“易户网”、企业服务“易企网”2个应用平台，统一办理居民和企业服务事项。力争市直部门50%以上行政审批事项，都能在网上办理或预约。推进政府工作项目化管理、责任化考核，促进政务服务提速增效。全面推进政务公开，行政决策过程和权力行使能公开的都及时公开。整合“市民心声”、市长热线、市长信箱等平台功能，完善“12345政府服务直通车”运行机制，对群众诉求做到件件有着落、事事有回音。提高运用法治思维、法律语言做好群众工作的能力，各级干部都要善于通过法律手段协调关系、化解矛盾、解决问题。

树立勤政廉洁政风。巩固和拓展群众路线教育实践活动成果，推动作风建设规范化、常态化、长效化。严肃工作纪律，端正工作作风。尤其要端正经济工作作风，坚决反对数字不实、盲目决策、乱铺摊子、过度负债、表面文章、不抓落实等不良风气。实施常态化公务员考核，整治庸政、懒政、怠政。深入开展“双联系”、“亲切服务”、政风行风评议等活动，真心实意践行“为民、务实、清廉”的要求。落实廉政建设责任制，扎实推进政府系统惩治和预防腐败体系建设，强化重点领域和关键环节的监管，健全用人、用钱、用地、用权制度，扎紧制度的笼子。强化效能监察和审计监督。严守中央八项规定和省、市相关规定，“三公”经费和财政供养人员只减不增。

各位代表！经济发展新常态对我们工作提出了诸多挑战，但也为芜湖实现“弯道超越”提供了难得机遇。多年来，芜湖坚持创新驱动、产业强市、统筹城乡不动摇，坚持一张蓝图干到底，打下了良好的发展基础。站在新的起点上，我们一定要以高度的历史责任感，在中共芜湖市委的坚强领导下，顺势而为，乘势而上，落实好人才优先的发展战略，奋力打造经济和城市两个升级版，当好皖江发展排头兵，为早日建成创新、优美、和谐、幸福新芜湖而不懈努力！
